# Reciprocal expression of Slug and Snail in human oral cancer cells

**DOI:** 10.1371/journal.pone.0199442

**Published:** 2018-07-03

**Authors:** Ryosuke Nakamura, Hiroki Ishii, Kaori Endo, Asami Hotta, Eiji Fujii, Keiji Miyazawa, Masao Saitoh

**Affiliations:** 1 Center for Medical Education and Sciences, Interdisciplinary Graduate School of Medicine, University of Yamanashi, Chuo, Yamanashi, Japan; 2 Department of Biochemistry, Interdisciplinary Graduate School of Medicine, University of Yamanashi, Chuo, Yamanashi, Japan; 3 Department of Oral and Maxillofacial Surgery, Kofu Municipal Hospital, Kofu, Yamanashi, Japan; Toho Daigaku, JAPAN

## Abstract

Snail, also called Snai1, is a key regulator of EMT. Snail plays crucial roles in cancer progression, including resistance to anti-tumor drugs and invasion by various cancer cells. Slug, also known as Snai2, is also involved in the aggravation of certain tumors. In this study, we examined the roles of Slug in human oral squamous cell carcinoma (OSCC) cells. Slug is highly expressed in these cells, and Slug siRNA effectively represses anti-tumor drug resistance and invasive properties. In addition, transforming growth factor (TGF)-β upregulates the expression of Snail and Slug and promotes resistance to anti-tumor drugs in OSCC cells. Surprisingly, Slug siRNA appears to upregulate Snail expression considerably in OSCC cells. Snail siRNA also appears to upregulate Slug expression. Thus, either Slug or Snail siRNA alone partially mitigates malignant phenotypes in the presence of TGF-β, whereas both Slug and Snail siRNAs together dramatically suppress them. Therefore, Slug and Snail in tandem, but not alone, are potential therapeutic targets for nucleic acid medicines to treat oral cancer.

## Introduction

The epithelial–mesenchymal transition (EMT) is an essential biological process during embryonic development, as well as during wound healing and tissue regeneration in adult tissues [1]. During embryonic development, EMT involves the complete loss of expression of epithelial marker proteins, including E-cadherin and keratins, in epithelial cells. Instead, the expression of mesenchymal marker proteins, including N-cadherin and vimentin, is induced to complete EMT [2,3]. However, the pathological significance of EMT in cancer remains controversial because partial, rather than complete, EMT is crucial for promoting invasion and metastasis [2,4]. It is clear, however, that EMT transcription factors (EMT-TFs) promote cancer progression by promoting invasion and drug resistance, but not tumorigenesis, as recently determined by numerous *in vitro* and *in vivo* studies using mouse cancer models [5–8]. The EMT-TFs include Twist, Snail, Slug, ZEB1 (a.k.a. δEF1), and ZEB2 (a.k.a. SIP1). The expression of these TFs is regulated transcriptionally and translationally by secreted factors, extracellular matrices, and exosomes in cancer cells [1]. The mRNA and protein levels of ZEBs correlate positively with the aggressive phenotypes and stem cell properties of breast cancer cells, whereas Snail protein, but not mRNA, was recently reported to be closely linked to them [9–11].

Snail, which is encoded by the *SNAI1* gene, and Slug, which is encoded by the *SNAI2* gene, are zinc-finger transcription factors belonging to the Snail family [12]. Both Snail and Slug are reportedly expressed in skeletal stem/stromal cells (SSCs) during the pre- and post-natal states. Moreover, targeting either Snail or Slug alone exerts only subtle effects on developmental programs, whereas simultaneous knockout of both markedly impairs SSC self-renewal, differentiation, and bone formation [13]. Thus, both proteins function redundantly during embryonic bone development in mice. In addition, the expression of both *SNAI1* and *SNAI2* is downregulated because their protein products occupy each other’s promoter during chondrogenesis, which provides an explanation for their genetic redundancy [14]. During EMT programs associated with development as well as cancer progression, Snail and Slug repress E-cadherin transcription by binding directly to E2 box–type elements (CAGGTG/CACCTG) found in its promoter [12]. Compared to the roles of Snail in EMT, those of Slug are not as well understood, particularly in cancer progression. Slug, which is aberrantly upregulated in pro–B cell acute leukemia, functions as an anti-apoptotic factor in normal hematopoietic progenitor cells [15]. Additionally, Slug specifically rescues hematopoietic progenitor cells from lethal doses of radiation [16]. Apart from blood cells, Slug is highly expressed in accordance with lymph node metastasis and poor survival in gastric cancer [17], and regulates the stemness status of colorectal cancer [18]. Thus, Slug as well as Snail is aberrantly expressed in some types of cancers and regulates many kinds of fundamental processes, including cell proliferation, apoptosis, and cell motility.

The roles of cytokines in EMT have been studied in many kinds of cancer cells [2]. Among these, transforming growth factor (TGF)-β is a well-known inducer of EMT, and often overexpressed in some cancer tissues [19]. Indeed, mice that lack TGF-β3, a TGF-β family member, exhibit a high frequency of cleft palate formation [20], probably due to absence of apoptosis and EMT in the medial edge epithelium during fusion of both upper jaws [21]. We previously reported that cancer cells express TGF-β abundantly in the bone-invading area as determined by immunohistochemical analyses using human specimens from oral cancer patients [22]. IL-6 is a multifunctional cytokine known to regulate immune and inflammatory responses [23]. Higher expression levels of IL-6 have also been observed in various human cancer tissues, and IL-6 is known to induce EMT through STAT3 activation in human breast cancer cells [24]. IL-8 is a pro-inflammatory chemokine identified as a potent neutrophil activator and chemotactic factor secreted from monocytes and macrophages [25]. Similarly to IL-6, IL-8 also promotes EMT and invasiveness through unknown mechanisms in lung cancer cells [26, 27].

In this study, we measured the expression of Slug in various oral squamous cell carcinoma (OSCC) cell lines. We found that Slug is highly expressed in OSCC cells, and that both Slug and Snail are upregulated by TGF-β. In addition, we found that Slug siRNA appears to cause an increase in Snail expression, whereas Snail siRNA appears to increase the level of Slug expression in these cells. Thus, the knockdown of Slug and Snail in tandem, but not either alone, efficiently suppresses invasive properties and chemo-resistance against anti-tumor drugs induced by TGF-β. These findings suggest that Slug and Snail, which are regulated reciprocally in cancer cells, are redundantly responsible for the malignant phenotype of oral cancer cells.

## Materials and methods

### Cell culture

Human oral squamous cell carcinoma (OSCC) cell lines (Ca9-22, HOC313, HSC2, HSC3, HSC4, OBC, OSC19, OSC20, OTC20, SAS, and TSU) were used in this study. Ca9-22, HSC2, HSC3, HSC4, and SAS were described previously [28]. HOC313, OBC, OSC19, OSC20, OTC20, and TSU were gifts from Dr. Yoshizawa (Oral and Maxillofacial Surgery, University of Yamanashi, Yamanashi, Japan). All cells were cultured in DMEM (Nacalai Tesque, Kyoto, Japan) supplemented with 4.5 g/L glucose, 10% FBS, 50 U/mL penicillin, and 50 μg/mL streptomycin at 37 °C under a 5% CO_2_ atmosphere.

### Reagents, antibodies, and plasmid construction

Recombinant human TGF-β1 was obtained from R&D Systems (Minneapolis, MN). Rabbit monoclonal anti–Slug, anti-STAT3, anti–phospho-STAT3 (705), and rat monoclonal anti-Snail antibodies were from Cell Signaling (Danvers, MA). Mouse monoclonal anti–α-tubulin and rat monoclonal anti–HA antibodies were from Sigma-Aldrich (St. Louis, MO). Docetaxel and Erlotinib were from Pepro Tech (Rocky Hill, NJ) and Wako (Osaka, Japan), respectively. The human Slug and human Snail expression plasmids were described previously [29].

### Immunoblot analysis

Cells were lysed in lysis buffer (20 mM Tris–HCl [pH 7.5], 150 mM NaCl, 1% Nonidet P-40, protease and phosphatase inhibitors). The protein concentration was measured using BCA protein assay reagent (Thermo Fisher Scientific, Waltham, MA). The harvested proteins separated by SDS-PAGE were transferred on to polyvinylidene difluoride membranes, followed by immunodetection with the ECL blotting system (GE Healthcare, Piscataway, NJ) on a Luminescent Image Analyzer (LAS400, Fujifilm, Tokyo, Japan).

### Quantitative real-time PCR (qRT-PCR)

Total RNA was extracted using the RNeasy mini kit (Qiagen, Venlo, Netherlands) and cDNAs were synthesized using the PrimeScript First Strand cDNA synthesis kit (TaKaRa Bio, Kusatsu, Japan). Quantitative RT-PCR analyses were performed using the Power SYBR Green PCR Master Mix (Applied Biosystems, Foster City, CA). The relative expression level of each mRNA was normalized using *GAPDH*. The following primers were used:

human Slug, forward, 5’-GCCTCCAAAAAGCCAAACTACA-3’, reverse, 5’-GAGGATCTCTGGTTGTGGTATGACA-3’;
human Snail, forward, 5’-TTCTCACTGCCATGGAATTCC-3’, reverse, 5’-GCAGAGGACACAGAACCAGAAA-3’;
human IL-6, forward, 5’-CCAGGAGCCCAGCTATGAAC-3’, reverse, 5’-CCAGGGAGAAGGCAACTG-3’;
human IL-8, forward, 5’-AAGGAAAACTGGGTGCAGAG-3’, reverse, 5’-ATTGCATCTGGCAACCCTAC-3’;
human GAPDH, forward, 5’-CGACCACTTTGTCAAGCTCA-3’, reverse, 5’-CCCTGTTGCTGTAGCCAAAT-3’.

### RNA interference

Transfection of siRNAs was performed in six-well tissue culture plates using Lipofectamine RNAiMAX transfection reagent (Invitrogen). The final concentration of siRNA was 10 nM. The Stealth RNAi siRNA sequences used in this study were as follows:

human Slug#1, 5’-CCGUAUCUCUAUGAGAGUUACUCCA-3’;
human Slug#2, 5’- GAUGCAUAUUCGGACCCACACAUUA-3’;
human Snail#1 5’-AGACCCACUCAGAUGUCAAGAAGUA-3’;
human Snail#2, 5’-CCUGUCAGAUGAGGACAGUGGGAAA-3’.

### Cell proliferation assay

Cells were seeded on six-well plates, reverse-transfected with the siRNAs, and cultured for 24 h. Cells were subsequently seeded in triplicate in 96-well tissue culture plates. After exposure to docetaxel for 24 h, cell count assays were carried out using Cell Count Reagent SF (Nacalai Tesque).

### Invasion assay

Transwell inserts with an 8 μm pore size (BD Falcon, Franklin Lakes, NJ) were coated with type I collagen gel (KOKEN, Tokyo, Japan). Suspended cells were seeded on the inner chamber and cultured for 24 h. Invaded cells were fixed and stained with Trypan Blue solution (Sigma-Aldrich) before being counted under an inverted microscope.

### Statistical analyses

The data are presented as the mean ± SD. Statistical analyses were performed using Student’s *t*-test between any two groups.

## Results

### Slug and Snail expression in OSCC cells

**Fig 1:**
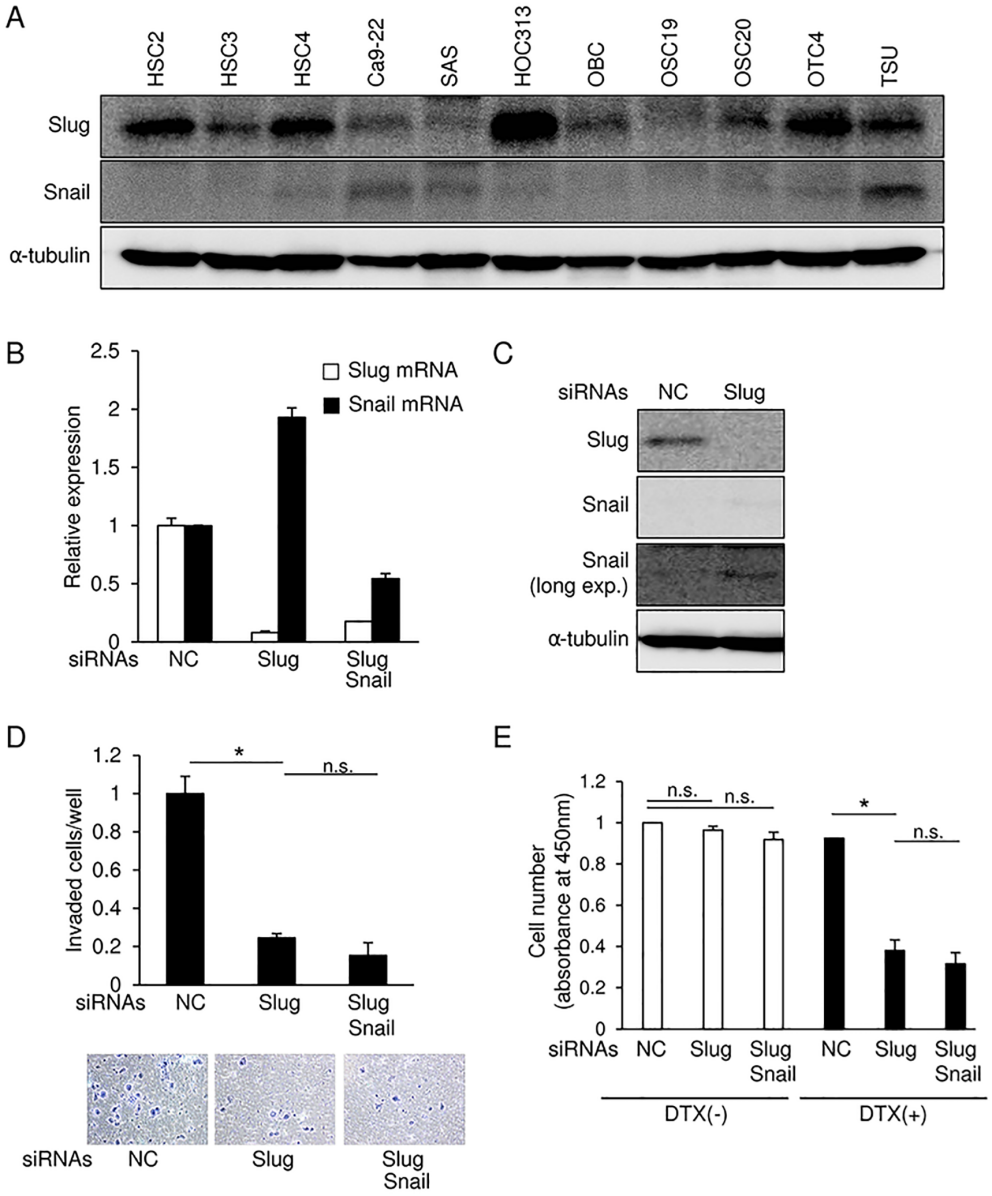
Slug and Snail expression in various OSCC cell lines. **(A)** Slug and Snail protein levels in OSCC cell lines were determined by immunoblotting with α-tubulin as a loading control. **(B and C)** Following the knockdown of either Slug alone, or of Slug and Snail in tandem, in HSC4 cells, mRNA and protein levels of Slug and Snail were examined by qRT-PCR (B) and immunoblot analysis (C), respectively. mRNA levels measured were normalized to the amount of *GAPDH* mRNA (B) while α-tubulin was used as a loading control for immunoblotting (C). long exp., long exposure. **(D)** Invasion assays were performed on HSC4 cells transfected with either Slug siRNA alone or both Slug and Snail siRNAs. After photos were taken (bottom panels), cell invasion was quantified (top panel). The value of the cells transfected with control siRNA is indicated as “1”. **(E)** After the knockdown of either Slug alone, or of Slug and Snail in tandem, in HSC4 cells, the cells were exposed to docetaxel (DTX; 3 μM) for 24 h. Cell viability was evaluated by cell count assay. The value of the control cells is indicated as “1”. NC, non-specific negative control siRNA. Slug siRNA (#1) and Snail siRNA (#1) were used. Each value represents the mean ± s.d. of triplicate determinations from a representative experiment. Similar results were obtained in at least three independent experiments *p* values were determined by Student’s *t-test*. *p < 0.05; n.s., not significant.

Snail is known to regulate EMT in various kinds of cancer cells and to protect some cells from cellular senescence in response to various stimuli [1]. Because of the similar primary structures of the Snail and Slug proteins, Slug is thought to have similar functions to those of Snail [14]. As our pilot studies, we observed that Slug mRNA is expressed at relatively high levels in head and neck cancer cells, compared to cancer cells from other tissues, whereas Snail mRNAs are ubiquitously expressed in the cells from almost all tissues according to qRT-PCR analyses (unpublished data). In addition, in cohort study of oral tongue squamous cell carcinoma (GSE75538), increased expression level of *SNAI2*, but not *SNAI1*, was found in OSCC tissues compared with that in adjacent normal tissues (S1 Fig). To determine specific roles of Slug in OSCC cells, we first determined Slug and Snail protein levels in several OSCC cells by immunoblotting (Fig 1A). Under the same experimental conditions, Slug protein was easily detected in most of the OSCC cell lines we tested, whereas Snail protein was not detected in some of them. Additionally, it appears that the cells that express Slug at high levels expressed lower levels of Snail. Next, we transfected HSC4 cells, in which Slug is expressed at relatively high levels, with siRNAs against Slug. Slug siRNAs successfully reduced Slug mRNA and protein levels (Fig 1B and 1C). Based on previous reports describing that, during bone development, expression levels of Snail and Slug are increased in the cells derived from Slug and Snail knockout mice, respectively (13,14), we sought to examine the expression of Snail and Slug in cancer cells upon transfection with the siRNAs. Slug siRNAs caused a slight increase in endogenous Snail mRNA and protein levels (Fig 1B and 1C). This finding was also confirmed in HOC313 cells (S2 Fig). Invasion assays in cells transfected with Slug siRNA showed suppressed invasion of HSC4 cells (Fig 1D), which is not significantly affected in combination with Snail siRNA. Similar to invasion assays, Slug knockdown significantly reduced chemo-resistance against docetaxel (DTX), a chemotherapeutic agent widely used in oral cancer patients, whereas combination of Snail siRNAs did not further affect it (Fig 1E). These findings suggested that, under normal culture conditions, Slug regulates invasiveness and chemo-resistance against anti-tumor drugs in HSC4 cells, and the increase in Snail levels following Slug knockdown may only negligibly affect these cellular phenomena.

### Upregulation of Slug and Snail in HSC4 cells following TGF-β treatment

**Fig 2:**
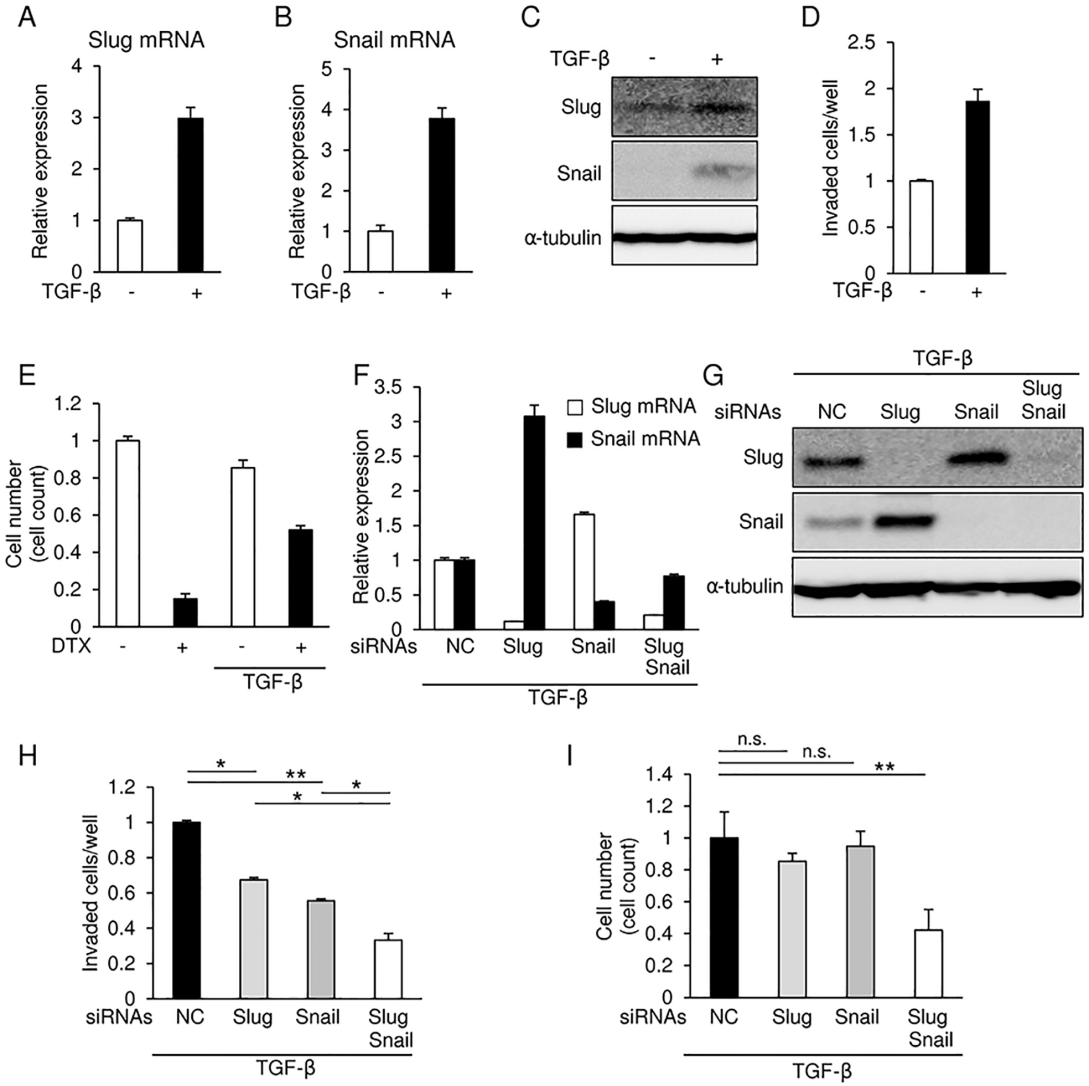
Slug and Snail induction in HSC4 cells in response to TGF-β. **(A, B, and C)** Slug and Snail mRNA and protein levels in HSC4 cells that had either been treated with 1 ng/ml TGF-β for 24 h or left untreated were determined by qRT-PCR (A and B) and immunoblot analyses (C), respectively. Values were normalized to the amount of *GAPDH* mRNA (A and B) while α-tubulin was used as a loading control for immunoblotting (C). **(D)** Invasion assays were performed in HSC4 cells treated with or without 1 ng/ml TGF-β, followed by quantification analyses. The value of the control cells is indicated as “1”. **(E)** HSC4 cells were treated with 1 ng/ml TGF-β for 24 h, the cells were exposed to docetaxel (DTX; 10 μM). The viable cells were trypsinized and counted using a hemocytometer. The value of the control cells is indicated as “1”. **(F and G)** Following the siRNA-mediated knockdown of either Slug, Snail, or both in HSC4 cells, the cells were treated with 1 ng/ml TGF-β for 24 h. The levels of Slug and Snail mRNA and protein were determined by qRT-PCR (F) and immunoblotting (G), respectively. mRNA levels were normalized to the amount of *GAPDH* mRNA (F) while α-tubulin was used as a loading control for immunoblotting (G). **(H)** After the siRNA-mediated knockdown of either Slug, Snail, or both in HSC4 cells treated with 1 ng/ml TGF-β, the cells were subjected to invasion assays, followed by taking photos and quantification. The value of the control cells is indicated as “1”. **(I)** After siRNA-mediated knockdown of either Slug, Snail, or both in HSC4 cells treated with 1 ng/ml TGF-β for 24 h, the cells were exposed to docetaxel (DTX) for 24 h. The viable cells were trypsinized and counted using a hemocytometer. The value of the control cells is indicated as “1”. Slug siRNA (#1) and Snail siRNA (#1) were used. Each value represents the mean ± s.d. of triplicate determinations from a representative experiment. Similar results were obtained in at least three independent experiments. NC, non-specific negative control siRNA. *p* values were determined by Student’s *t-test*. *p < 0.05, **p < 0.01; n.s., not significant.

We previously reported that cancer cells express TGF-β, a key inducer of the EMT, abundantly in the bone-invading area in human specimens from oral cancer patients [22]. It is well known that repression of E-cadherin, a representative EMT marker, is frequently observed in the cells only at the invasion front, but not the center of tumor, suggesting that TGF-β regulates EMT in this region in vivo. We thus examined expression of Slug and Snail in the presence of TGF-β. TGF-β caused an increase in the levels of both Slug and Snail mRNA and protein in HSC4 cells (Fig 2A, 2B and 2C). In addition, TGF-β increased the number of invaded cells as determined by invasion assays in HSC4 cells (Fig 2D). Just as previous reports have indicated that TGF-β increases chemo-resistance towards various anti-tumor drugs in many kinds of cancer cells [30], TGF-β increased chemo-resistance to DTX in HSC4 cells (Fig 2E). siRNAs against Slug successfully silenced their endogenous target mRNA even in the presence of TGF-β, and also caused a considerable upregulation of Snail expression (Fig 2F and 2G).

Interestingly, when Snail upregulated by TGF-β was silenced by its specific siRNA, the level of both Slug mRNA and protein increased slightly in HSC4, SAS, and HOC313 cells (Fig 2F and 2G and S2 Fig). Indeed, the invasiveness induced by TGF-β was considerably inhibited by either siRNA alone, and further inhibited by a combination of Slug and Snail siRNAs (Fig 2H). In addition, chemo-resistance to DTX induced by TGF-β was not significantly affected by either siRNA alone, but dramatically reduced by combined transfections with both siRNAs in HSC4 cells (Fig 2I) and SAS cells (S2 Fig). Thus, these findings indicate that aggressive cellular phenotypes, particularly chemo-resistance, induced by TGF-β are ameliorated by the knockdown of Snail and Slug in tandem, but not of either alone. Moreover, these findings suggest that, in the presence of TGF-β, Slug and Snail together regulate invasiveness and chemo-resistance in OSCC cells.

### Enhancement of chemo-resistance towards anti-tumor drugs in SAS cells overexpressing either Slug or Snail

**Fig 3:**
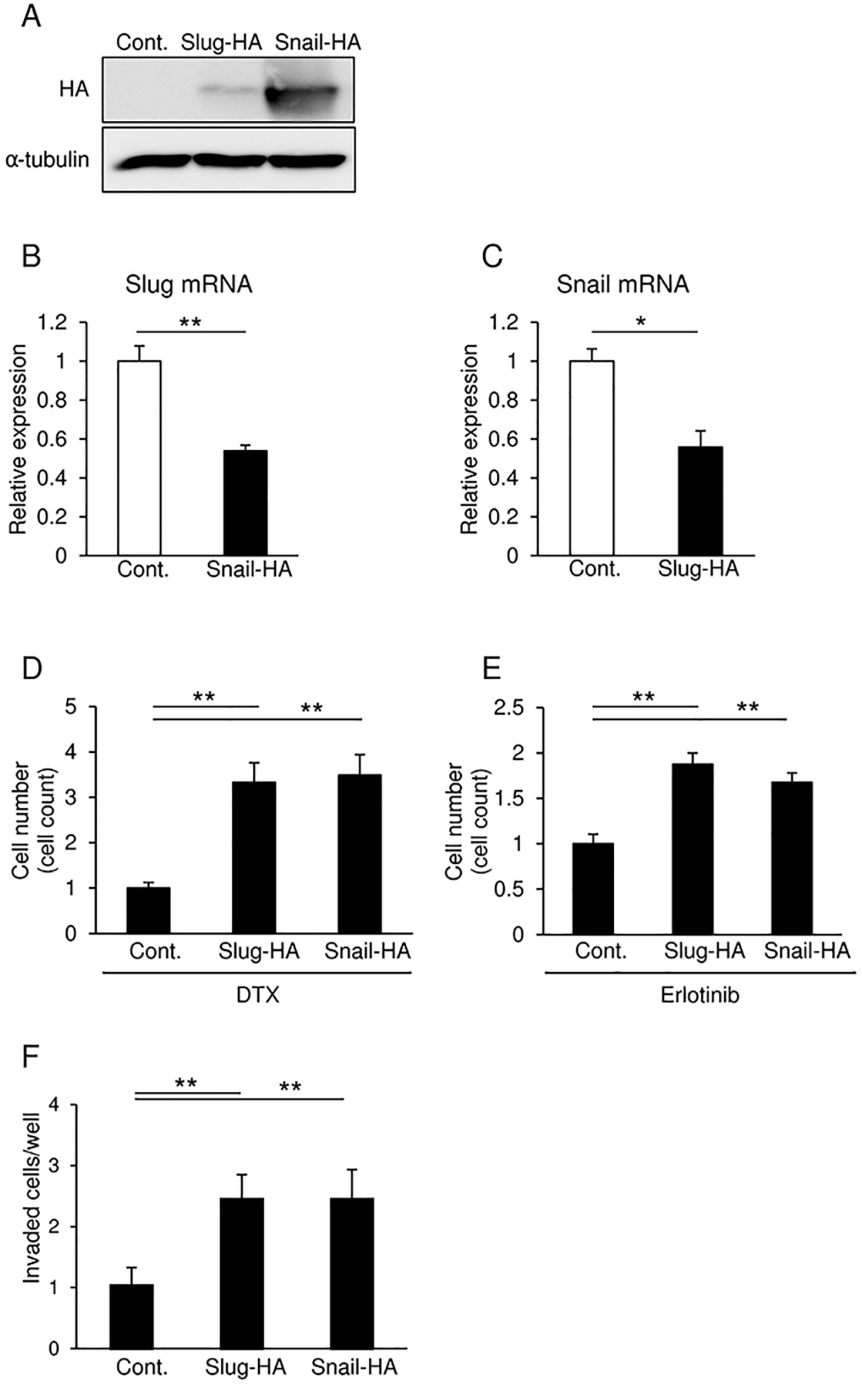
Overexpression of Slug and Snail in SAS cells. **(A, B, and C)** SAS cells transfected with plasmids encoding either HA-tagged Slug or Snail were subjected to immunoblot (A) and qRT-PCR analyses (B and C). α-tubulin was used as a loading control (A). mRNA levels were normalized to the amount of *GAPDH* mRNA (B and C). **(D and E)** SAS cells transfected with plasmids encoding either HA-tagged Slug or Snail were exposed to docetaxel (DTX; 10 μM) (D) or Erlotinib (5 μM) (E) for 24 h. The viable cells were trypsinized and counted using a hemocytometer. The value of the control cells is indicated as “1”. **(F)** Invasion assays were performed on SAS cells transfected with either HA-tagged Slug or Snail. The value of the control cells is indicated as “1”. Each value represents the mean ± s.d. of triplicate determinations from a representative experiment. Similar results were obtained in at least three independent experiments. Cont., negative control plasmid. *p* values were determined by Student’s *t-test*. **p < 0.01.

Since SAS cells exhibited better transient transfection efficiency than HSC4 cells (data not shown), expression plasmids encoding HA-tagged full-length human Slug or Snail were transfected into the cells. After transfection, Slug protein levels were lower compared to those of Snail (Fig 3A). By contrast, we detected increases of approximately 8- and 2.5-fold in the mRNA levels of transfected-Slug and -Snail, respectively. These results were obtained with almost similar transfection efficiency for the Slug and Snail expression plasmids as determined by immunohistochemistry using anti-HA antibody (data not shown), suggesting that the levels of ectopically expressed Snail protein are more stable than those of Slug in the transfected cells. However, Slug overexpression was sufficient to significantly reduce endogenous Snail mRNA, and vice versa (Fig 3B and 3C). Next, we examined chemo-resistance against anti-tumor drugs, such as DTX or Erlotinib, a small molecular agent that specifically targets EGFR tyrosine kinase, in SAS cells overexpressing either Slug or Snail. Overexpression of either Slug or Snail increased invasive properties and the number of viable cells in response to treatment with both anti-tumor drugs (Fig 3D, 3E and 3F). These findings suggest that overexpression of Slug or Snail regulates mutual expression, and enhances chemo-resistance against anti-tumor drugs in SAS cells.

### IL6 and IL8 mRNA levels in OSCC cells transfected with siRNAs against Slug or Snail

**Fig 4:**
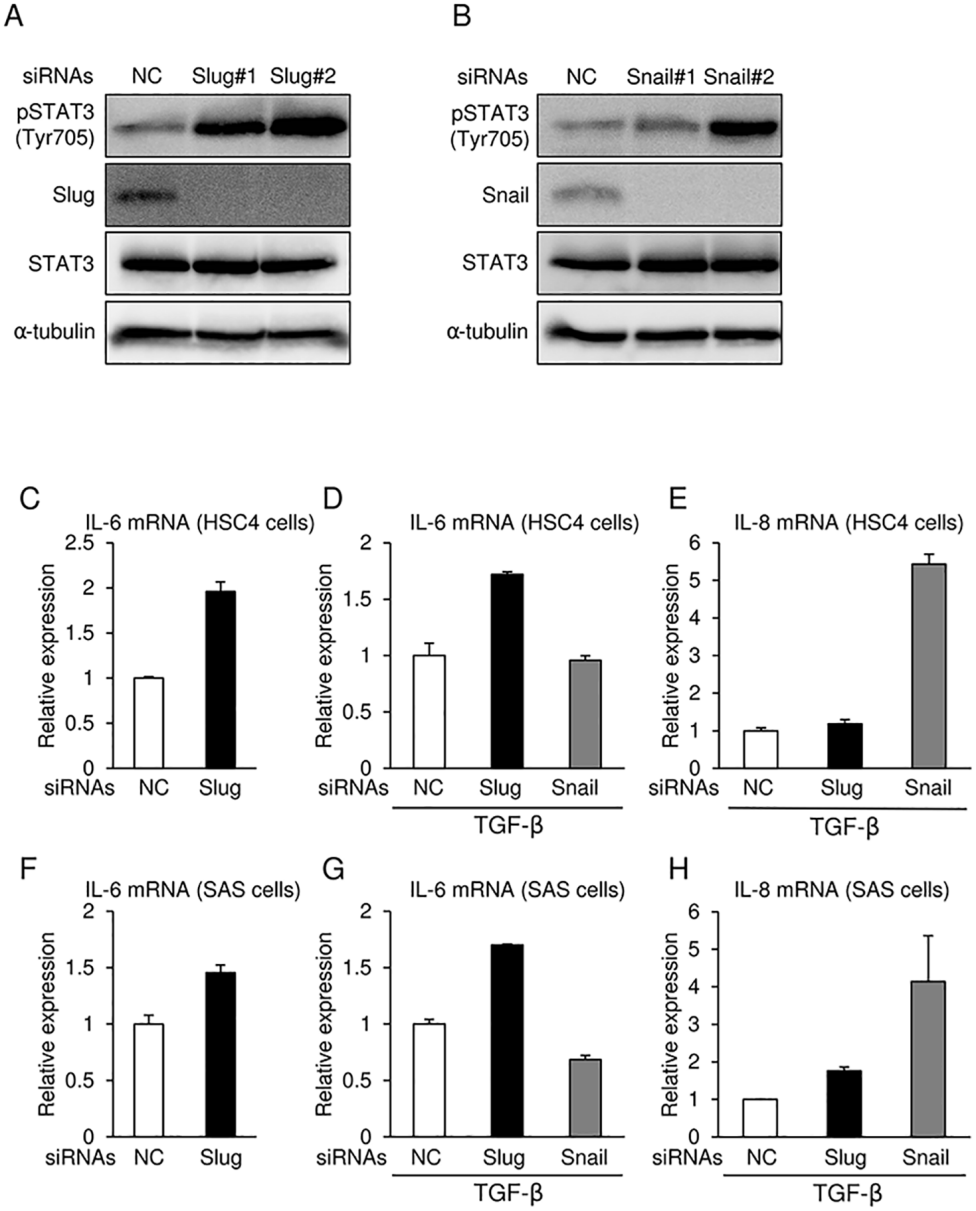
Slug and Snail siRNAs regulate mRNA expression of IL-6 and IL-8. **(A and B)** Phosphorylation of STAT3 at Y705 residue were determined by immunoblotting following transfection with either Slug or Snail siRNA in SAS cells treated with TGF-β. α-tubulin was used as a loading control. **(C–H)** After the knockdown of either Slug or Snail in HSC4 (C, D, and E) and SAS (F, G, and H) cells, the cells were treated with 1 ng/ml TGF-β for 24 h. mRNA levels of IL-6 (C, D, F, and G) and IL-8 (E and H) were analyzed by qRT-PCR. mRNA levels were normalized to the amount of *GAPDH* mRNA. Slug siRNA (#1 and #2) and Snail siRNA (#1 and #2) were used. Each value represents the mean ± s.d. of triplicate determinations from a representative experiment. Similar results were obtained in at least three independent experiments. NC, non-specific negative control siRNA.

To determine the mechanism by which Slug and Snail siRNAs upregulate Snail and Slug, respectively, in human OSCC cells, we examined several signaling molecules including AKT, ERK, p38MAPK, JNK, NF-κB/IκB, HMGA2, and STAT3, which are reported to regulate transcription of Snail, by immunoblot analyses with specific antibodies and anti-phospho antibodies. Of these, STAT3 phosphorylation at tyrosine 705 was increased by both Slug and Snail siRNAs in SAS cells (Fig 4A and 4B). STAT3 phosphorylation is inhibited by inhibitory molecules for STAT3, PIAS3, and SOCS3, but their expression levels were not significantly changed by both siRNAs (data not shown). Next, we performed qRT-PCR and analyzed the mRNA levels of several cytokines and growth factors. Among them, IL-6 was increased by Slug siRNA in the absence or presence of TGF-β in HSC4 and SAS cells (Fig 4C, 4D, 4F and 4G). In addition, Snail siRNA also upregulated IL-8 expression in both cells treated with TGF-β, but not in cells that were not treated with TGF-β (Fig 4E and 4H, and data not shown). Thus, Slug siRNA and Snail siRNA enhanced STAT3 phosphorylation probably due to the upregulation of both IL-6 and IL-8, which regulate each other’s expression. Therefore, the combination of siRNAs targeting both Snail and Slug, rather than either alone, would be much more useful for nucleic acid medicine to treat human oral cancer patients.

## Discussion

In this study, we found that Slug protein levels are relatively high in human OSCC cells, and that cells in which Slug expression has been silenced exhibit increased sensitivity to anti-tumor drugs and reduced motile properties. Thus, Slug may play important roles in cancer progression in human OSCC cells. On the other hand, TGF-β is thought to be a well known inducer of EMT in cancer cells at invasion front of cancer tissues [31]. Under conditions of TGF-β stimulation, Slug and Snail were upregulated in human OSCC cells, as previously reported [32]. Molecular mechanisms of TGF-β–induced Snail expression have been proposed in several reports; Snail is directly upregulated by the TGF-β–Smad pathway in mouse normal mammary gland epithelial NMuMG cells, as demonstrated by treatment with cycloheximide [33]. HMGA2, induced by the TGF-β–Smad pathway, increases Snail expression in the same cells, indicating that Snail is also an indirect target for the TGF-β–Smad pathway [34]. Taken together, Snail induction by TGF-β in NMuMG cells is rapidly upregulated by Smad pathway and sustained by HMGA2, leading to the biphasic manner of the cellular response to TGF-β. In addition, we found that TGF-β–Smad pathway engages in crosstalk with the STAT3 pathway in cancer cells harboring a *KRAS* mutation [35]. However, the molecular mechanisms of TGF-β–induced Snail expression are not fully understood in OSCC cells. Interestingly, we found that Slug knockdown increases Snail expression and vice versa. Snail and Slug function redundantly in various kinds of cells and reciprocal regulation of gene expression between them is observed in at least palate, bone and cardiac formation. These compensatory regulation of Snail and Slug could be indispensable for EMT in embryonic development as well as cancer progression. However, the underlying molecular mechanism has not been elucidated yet, but the involvement of miRNA has not been ruled out in these processes. miR-34 and Snail negatively regulate each other [36], as do miR-203 and Slug [37]. Thus, it is possible that Slug and Snail downregulate miR-34 and miR-203, respectively, in OSCC cells. Furthermore, we found weak negative correlation between *SNAI1* and *SNAI2* expression in cohort study of oral tongue squamous cell carcinoma (S1 Fig). Taken together, our findings suggest that, in terms of nucleic acid medicine, silencing only one of the two genes is not sufficient for chemo-resistance against anti-tumor drugs and invasiveness in OSCC cells. Snail family–targeted therapy for oral cancers will require the development of anti-tumor drugs that target both proteins simultaneously.

siRNAs against Slug and Snail upregulate levels of IL-6 and IL-8, cytokines known to enhance STAT3 phosphorylation and to be involved in EMT. However, it is unclear how siRNAs against Slug and Snail upregulate *IL6* and *IL8* mRNA, and promote STAT3 phosphorylation. Since the expression of SOCS3 and PIAS3, negative regulators for STAT3, was not altered by either siRNA (data not shown), STAT3 activation is dependent on autonomous IL-6 or IL-8 secretion. Additionally, we found that the STAT3 inhibitor, Stattic, repressed the induction of Snail by Slug siRNA and Slug by Snail siRNA (data not shown). Stattic also inhibited Slug expression even without Snail siRNA, suggesting that STAT3 activation is fundamentally required for Slug expression in OSCC cells. However, the phosphorylation levels of STAT3 were not completely consistent with the expression levels of Slug in OSCC cells used in Fig 1A (data not shown), which suggests a requirement for the additional signals. Therefore, Slug and Snail could reciprocally regulate each other’s expression probably through, at least in part, STAT3 activation induced by autonomously secreting IL-6 and IL-8.

Snail undergoes post-transcriptional modifications through the consensus phosphorylation motif of GSK-3β [38]. As in the case of β-catenin, GSK-3β–mediated phosphorylation in Snail provokes its cytoplasmic export and subsequent ubiquitin-mediated proteasome degradation by β-TrCP [38]. Recently, it is reported that GSK-3β–mediated degradation is inhibited by the binding of A20, also known as TNFAIP3, to Snail [11]. A20 monoubiquitinates Snail at three lysine residues at its C-terminus, which fails to associate with and be phosphorylated by GSK-3β, leading to the stabilization of Snail [11]. Although these phosphorylation sites are not conserved in Slug, other post-translational modifications of Slug could exist to regulate transcription of the genes that mediate cancer progression. Indeed, Slug protein levels do not always reflect its mRNA levels in OSCC cells (Fig 1A and data not shown). Moreover, overexpression of Snail enhances expression of ZEB1, another key molecule for EMT induction, and in turn promotes the aggressiveness of cancer cells [39]. Previous reports indicated that the upregulation of ZEB1 by TGF-β or other cytokines/growth factors is accompanied by ZEB2 upregulation [40], and that both ZEB1 and ZEB2 are highly expressed in breast cancer cells with aggressive phenotypes [9]. Because ZEB1 and ZEB2 are reportedly to be reciprocally controlled by TGF-β in endothelial cells [41], the simultaneous knockdown of ZEB1 and ZEB2, rather than knockdown of either alone, would be useful for potential therapeutic strategy, as previous report [33,42]. Therefore, diagnosis and therapy, which target EMT-TFs, including those of the Snail and ZEB families, will require the development of methods that can recognize both proteins simultaneously as well as anti-tumor drugs that can target both proteins simultaneously. Therefore, EMT-TFs regulate their function at both transcriptional and post-translational levels by other member of EMT-TFs, leading to a sophisticated machinery of EMT induction and cancer progression.

## Supporting information

S1 Fig *SNAI1* and *SNAI2* expression in oral tongue squamous cell carcinoma. (A) SNAI1 and SNAI2 expression levels in noncancerous (adjacent normal tissuues) and cancerous tissues from OSCC patients (n = 14). (B) Correlation between SNAI1 and SNAI2 expression in cancerous tissues from OSCC patients (n = 14). Publicly available dataset from OSCC patients (GSE75538) was used.
(PDF)

S2 Fig Slug and Snail expression in HOC313 and SAS cells. (A) After knockdown of only Slug alone or of both Slug and Snail tandem in HOC313 cells, mRNA levels of Slug and Snail were examined by qRT-PCR. (B, C, D, and E) After transfection with the indicated siRNAs in SAS (B and C) and HOC313 (D and E) cells, the cells were treated by 1 ng/ml TGF-β for 24 h. Slug and Snail mRNA and protein levels were determined by qRT-PCR (B and D) and immunoblot analysis (C and E), respectively. Values were normalized to the amount of GAPDH mRNA (A, B, and D). α-tubulin was used as a loading control (C and E). (F) After transfection with the indicated siRNAs, SAS cells were exposed to docetaxel (DTX; 10 μM) for 24 h. The viable cells were trypsinized and counted using a hemocytometer. The value of the control cells is indicated as “1”. NC, non-specific negative control siRNA. Slug siRNA (#1) and Snail siRNA (#1) were used. p values were determined by Student’s t-test. ***p < 0.001; n.s., not significant.
(PDF)

## Abbreviations

EMT: epithelial–mesenchymal transition
EMT-TF: EMT transcription factor
OSCC: oral squamous cell carcinoma
qRT-PCR: quantitative RT-PCR
siRNA: short interfering RNA
TGF-β: transforming growth factor-β
