# Supplementary figures and images for: Reciprocal expression of Slug and Snail in human oral cancer cells
Source: PLoS One. 2018 Jul 3;13(7):e0199442. doi: 10.1371/journal.pone.0199442 (PMC6029773; doi:10.1371/journal.pone.0199442)

S1 Fig. *SNAI1* and *SNAI2* expression in oral tongue squamous cell carcinoma.

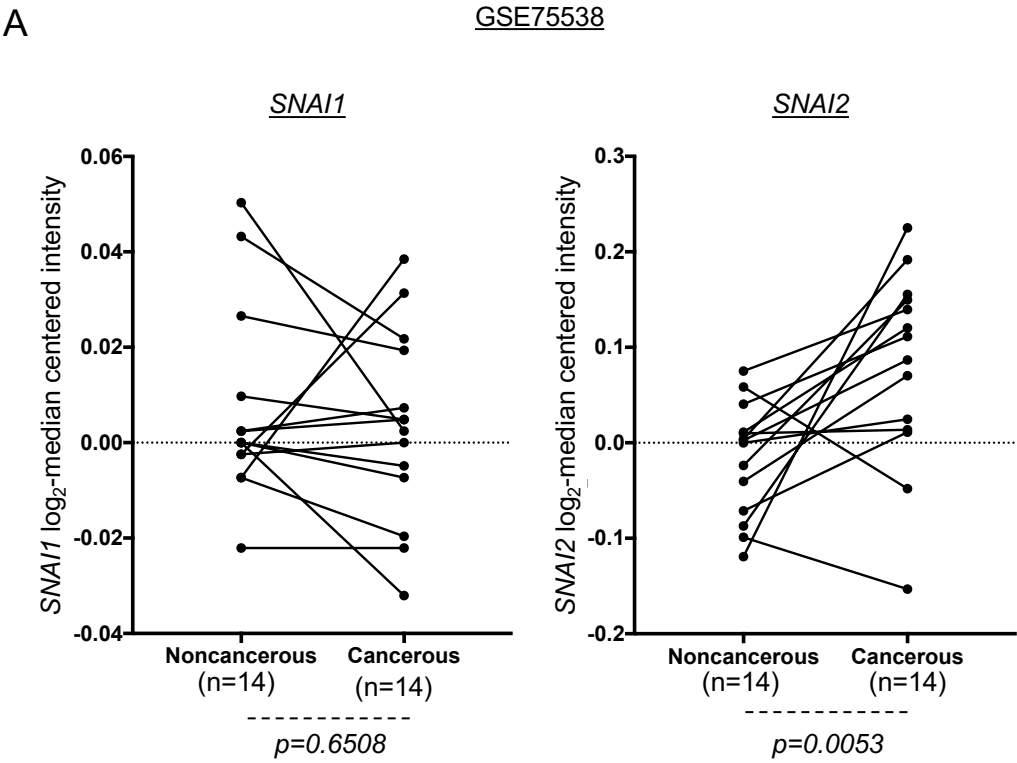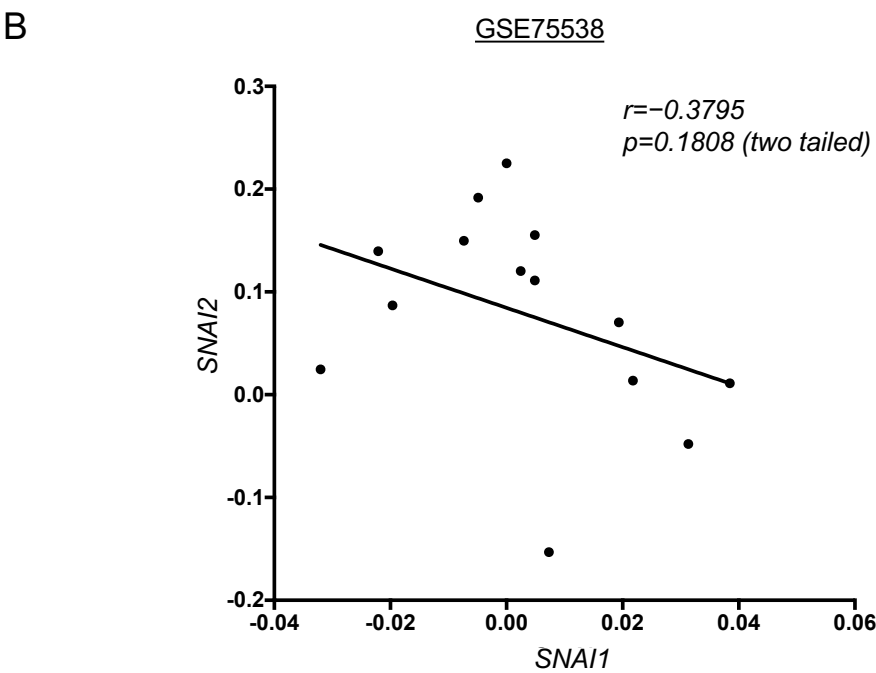

Supplement: S1 Fig — (A) SNAI1 and SNAI2 expression levels in noncancerous (adjacent normal tissuues) and cancerous tissues from OSCC patients (n = 14). (B) Correlation between SNAI1 and SNAI2 expression in cancerous tissues from OSCC patients (n = 14). Publicly available dataset from OSCC patients (GSE75538) was used. (PDF) [file pone.0199442.s001.pdf]

## S2 Fig. Slug and Snail expression in HOC313 and SAS cells.

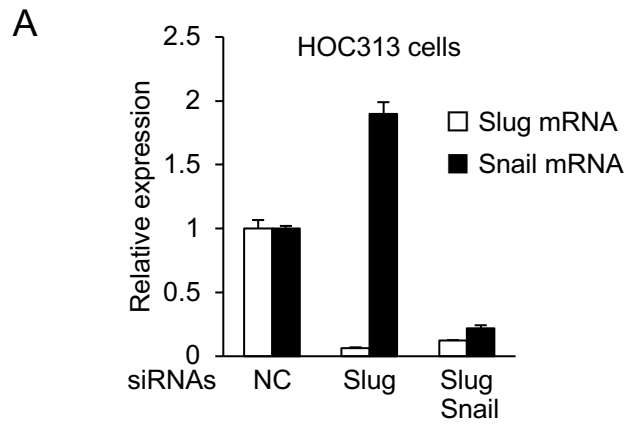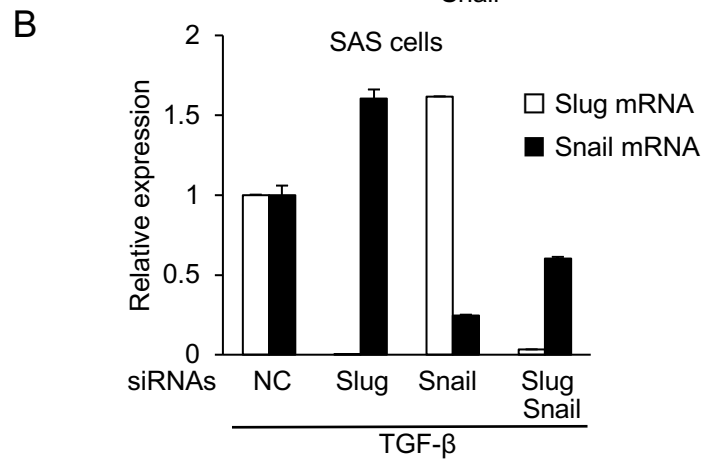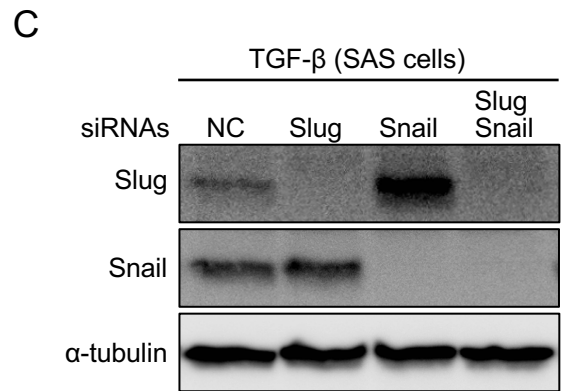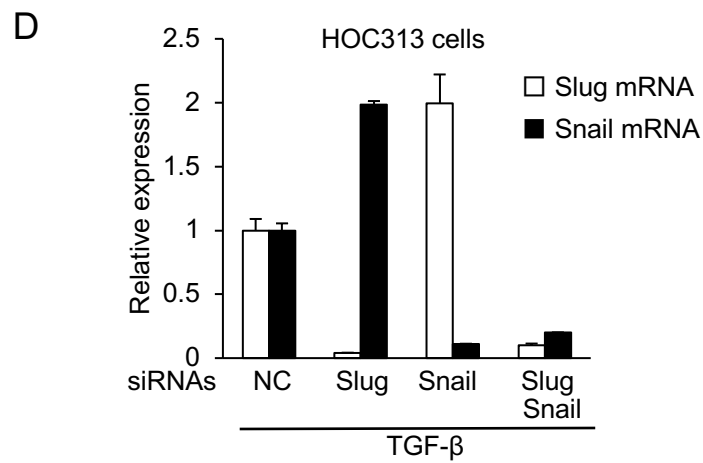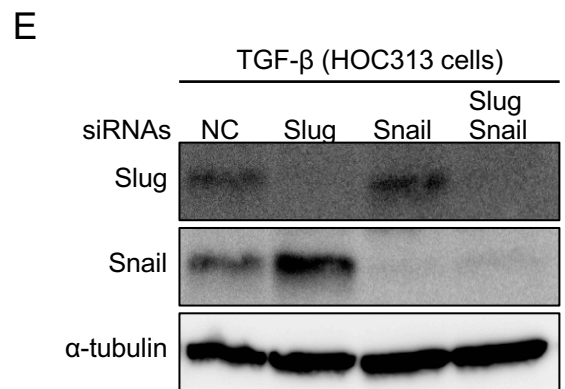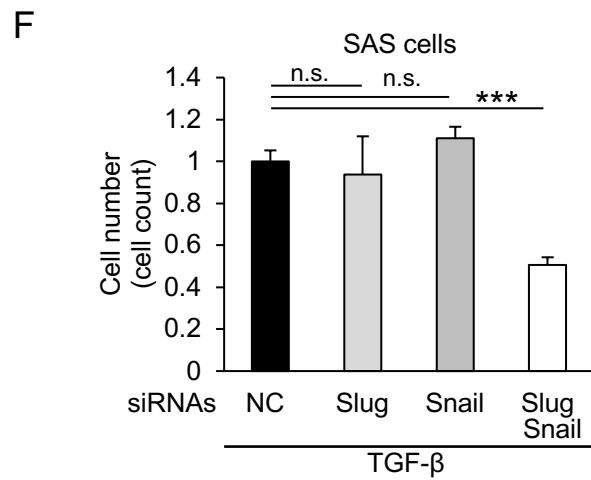

Supplement: S2 Fig — (A) After knockdown of only Slug alone or of both Slug and Snail tandem in HOC313 cells, mRNA levels of Slug and Snail were examined by qRT-PCR. (B, C, D, and E) After transfection with the indicated siRNAs in SAS (B and C) and HOC313 (D and E) cells, the cells were treated by 1 ng/ml TGF-β for 24 h. Slug and Snail mRNA and protein levels were determined by qRT-PCR (B and D) and immunoblot analysis (C and E), respectively. Values were normalized to the amount of GAPDH mRNA (A, B, and D). α-tubulin was used as a loading control (C and E). (F) After transfection with the indicated siRNAs, SAS cells were exposed to docetaxel (DTX; 10 μM) for 24 h. The viable cells were trypsinized and counted using a hemocytometer. The value of the control cells is indicated as “1”. NC, non-specific negative control siRNA. Slug siRNA (#1) and Snail siRNA (#1) were used. p values were determined by Student’s t-test. ***p < 0.001; n.s., not significant. (PDF) [file pone.0199442.s002.pdf]
